# Novel Multi-IMU Tight Coupling Pedestrian Localization Exploiting Biomechanical Motion Constraints

**DOI:** 10.3390/s20185364

**Published:** 2020-09-18

**Authors:** Dina Bousdar Ahmed, Estefania Munoz Diaz, Juan Jesús García Domínguez

**Affiliations:** 1 German Aerospace Center (DLR), Institute of Communications and Navigation, 82234 Oberpfaffenhofen, Germany; estefania.munoz@dlr.de; 2 Electronics Department of the University of Alcalá, 28805 Alcalá de Henares, Spain; jjesus.garcia@uah.es

**Keywords:** fusion, model, heading, experiment, wearables, inertial navigation, step and heading, strapdown, parameter estimation, evaluation, ground truth

## Abstract

In this article, we present a novel tight coupling inertial localization system which simultaneously processes the measurements of two inertial measurement units (IMUs) mounted on the leg, namely the upper thigh and the front part of the foot. Moreover, the proposed system exploits motion constraints of each leg link; that is, the thigh and the foot. To derive these constraints, we carry out a motion tracking experiment to collect both ground truth data and inertial measurements from IMUs mounted on the leg. The performance of the tight coupling system is assessed with a data set of approximately 10 h. The evaluation shows that the average 2D-position error of the proposed tight coupling system is at least 50% better than the average 2D-position error of two state-of-the-art systems, whereas the average height error of the tight coupling system is at least 75% better than the average height error of the two state-of-the-art systems. In this work, we improve the accuracy of the position estimation by introducing biomechanical constraints in an inertial localization system. This article allows to observe, for the first time, heading errors of an inertial localization system by using only inertial measurements and without the need for using maps or repeating totally or partially the walked trajectory.

## 1. Introduction

The safety of first responders like firefighters or police officers during emergency responses is key to guarantee their success in keeping us all safe. To this day, the safety of first responders relies mainly on strict operation protocols. In some cases, technology enables added functionality, for example, radio communication systems for firefighters [1] or monitoring of heart rate, temperature and humidity [2].

There is one important challenge that technology does not cover yet: accurate indoor localization of first responders during emergency responses in challenging environments, for example, environments with poor reception of satellite signals or fragile infrastructure. A potential solution is the use of inertial technology [3], which can also be used to predict the user’s activity [4], which is an added value of this technology. For instance, we can use inertial localization systems to detect if a firefighter collapsed during the emergency response.

Inertial localization has multiple challenges; among which the most characteristic one is the heading drift [5,6]. The heading is the direction of the user’s walk and the heading drift is the error accumulation in the heading [7], which occurs due to the integration of the errors in the turn rate measurements during the estimation of attitude of the IMU.Such error accumulation is a challenge common to all inertial localization systems [5,6] and affects the accuracy of the estimated odometry, that is, the position solution. There are different techniques that target the mitigation of the effect of the heading drift in the accuracy of the position estimation. We refer to these techniques as drift-compensation techniques and we distinguish two categories:

- drift compensation based on algorithms. In this category, inertial localization systems use techniques like simultaneous localization and mapping (SLAM) [8,9] or landmark detection [10,11,12] to reduce the drift in the odometry estimation.
- drift compensation based on sensor fusion. In this category, inertial localization systems incorporate information from other sensors, for example, global navigation satellite system (GNSS) measurements [13,14,15] or WiFi measurements [10,16] to correct the position solution of the inertial localization system.

In this work, we focus on drift-compensation techniques based on sensor fusion. More specifically, we focus on the combination of the measurements from different IMUs. We refer to these as multi-IMU localization systems. One approach that exploits multiple IMUs for inertial localization is to create an IMU array and average the raw measurements. For instance, Skog etal show that the noise in the accelerometer measurements can be reduced by averaging the measurements of the sensors placed on a 2D-array [17]. The authors of Reference [18] propose different algorithms to combine up to five IMUs. Each of the proposed algorithms combines the raw measurements of the IMUs in a different way and the authors analyze the benefits and shortcomings of each algorithm.

There are different works that use multiple IMUs and incorporate motion constraints. For instance, Meng etal use seven IMUs distributed around both legs to estimate the step length [19]. The authors propose a leg model that incorporates not only the attitude of each leg limb, but also hard constraints on some of the attitude angles. Another example can be found in Reference [20], where the authors place an IMU on each foot. The proposed algorithm estimates two different position estimations with the raw measurements form each respective IMU. Then, the authors apply a constraint on the maximum separation allowed between both position estimations. This constraint is based on the physiological limitations of a pedestrian.

We have noticed that the state-of-the-art does not guarantee that the attitude estimation of an inertial localization system is coherent with respect to human motion. In this work, we use the term coherent motion of a body limb to refer to the motion that is expected from this body limb. Such motions have been extensively studied and characterized in medicine [21]. For instance, Skog etal do not take into account the motion of the limbs during the walk [20]. Moreover, Meng etal impose hard constraints on the motion of the limbs which do not model the limb motion that the IMU is measuring [19].

Our study in Reference [22] shows that the error accumulation in an inertial localization system leads to the estimation of an incoherent motion of the limb on which the IMU is mounted. In Reference [22], we have studied two independent single-IMU localization systems. More specifically, a localization system based on a thigh-mounted IMU and a localization system based on a foot-mounted IMU.

**Figure 1:**
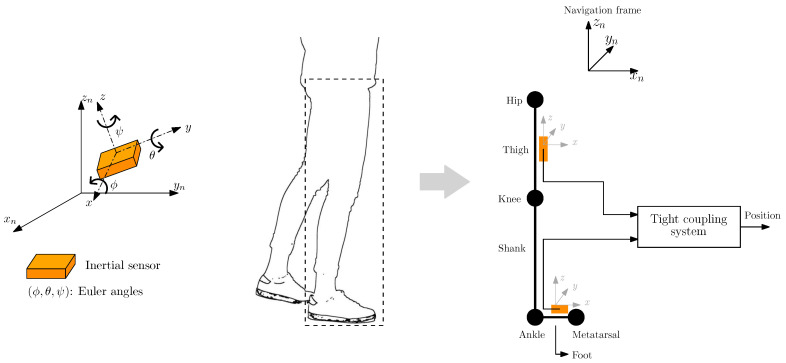
(**Left**) Representation of the navigation frame $\left(x_{n},y_{n},z_{n}\right)$, which is fixed, and the IMU frame (x,y,z). (**Right**) Representation of the human leg as a set of joints, namely the hip, knee, ankle and metatarsal, and links, namely the thigh, shank and foot. The IMUs are indicated by the orange boxes. This figure exemplifies the mounting of the IMUs on the human leg and how the tight coupling system uses the measurements of both sensing devices simultaneously to estimate the pedestrian’s position.

The overall goal of this article is to propose a tight coupling system for pedestrian localization based on the fusion of the inertial measurements from a thigh-mounted IMU and a foot-mounted IMU, as Figure 1 indicates. Our proposed system exploits the motion constraints of the human leg. We have the following objectives:

- Analyze the motion constraints of the human leg and formulate them in terms of the Euler angles of both the upper thigh and the foot.
- Propose a tight coupling system for pedestrian localization which combines the inertial measurements of a thigh-mounted IMU and a foot-mounted IMU and integrates motion constraints of both leg links, namely the upper thigh and the foot.
- Evaluate the effect of the motion constraints in the estimation of the Euler angles of the tight coupling system.
- Evaluate the performance of the tight coupling system and compare it to the performance of state of the art systems.

## 2. Research Methodology

In this section, we describe in detail our tight coupling system which incorporates the biomechanical dimension into the position estimation process. Firstly, we analyze the motion of the leg in order to understand how a person moves and the limitations of human physiology. Then, we study how the errors in an inertial localization system translate into the leg motion. Secondly, we integrate the outcome of the aforementioned analysis in the tight coupling system. One of our main considerations in to avoid the use of hard constraints in our tight coupling system, as opposed to Reference [19].

### 2.1. Background: Motion Tracking Experiment

In order to analyze the motion of the leg, we carry out an experiment to simultaneously measure:

- Inertial data, that is, acceleration and turn rate, of each of the leg links of one leg. The inertial data is measured with an inertial sensor, more specifically a MTw from Xsens [23].
- Ground truth, that is, position and attitude, of each of the leg links of one leg. The ground truth is measured with a camera-based motion tracking system (https://www.vicon.com/file/vicon/bonita-brochure.pdf) which tracks continuously over time the position and attitude of predefined objects.

**Figure 2:**
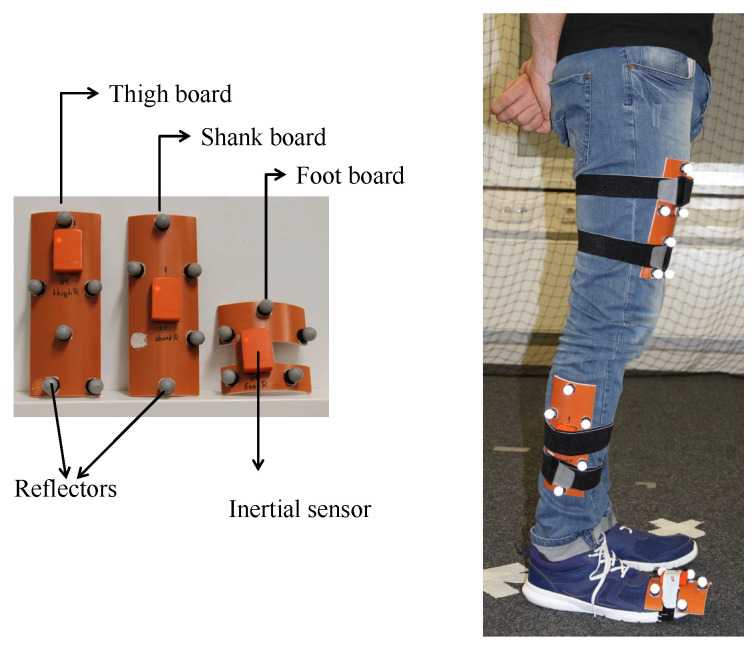
(**Left**) Mounting frame prepared for the experiments. On each mounting frame, we mounted an inertial sensor (orange boxes) and a set of reflectors which uniquely identify each mounting frame. (**Right**) Set up of the mounting frame on a volunteer’s leg. In this work, we only make use of the data from the thigh and the foot mounting frames.

**Figure 3:**
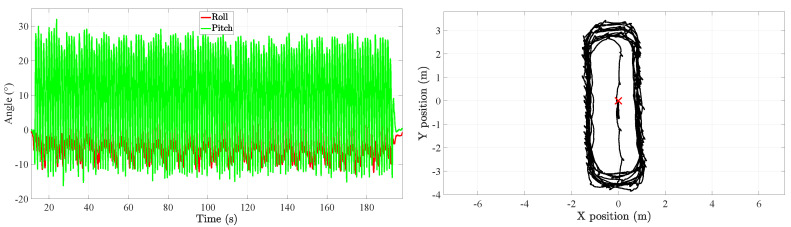
(**Left**) Tilt angles, i.e., roll and pitch, associated to the rectangle trajectory. (**Right**) Example of a rectangle trajectory during the motion tracking experiment. The red cross at (0,0) indicates the start and end of each walk.

The experiments here presented have also been used in Reference [22]. During these experiments, the volunteers were equipped with the mounting frames presented in Figure 2. Although it shows that the volunteer wears three mounting frames, namely on the thigh, shank and foot; we only make use of the thigh and foot information in this work. The volunteers performed three different types of trajectories in an 8 m × 3 m area: a rectangle trajectory, an eight-shape trajectory and a random trajectory. Moreover, each trajectory was repeated twice. The experiments are summarized in Table 1. A total of nine volunteers of different heights and weights participated in the experiment. In total, we have more than 3 h of data, that is, the inertial measurements and the true position and attitude of each leg link. An example is shown in Figure 3 of the Euler angles and the associated rectangle trajectory.

An important aspect of our experiment is the synhronization of the inertial data with the ground truth data. In order to synchronize these two types of information, we asked each volunteer to perform a synchronization sequence at the beginning and end of each walk. By means of this synchronization sequence, we could synchronize the inertial data with the ground truth data and therefore compare them.

In the following, we analyze the motion of the legs in two parts—the tilt angles, that is, the roll and pitch, and the heading difference between the thigh and the foot. The reason for this approach is that the heading is not observable with inertial measurements [24]. Therefore, we study the heading of one limb with respect to the heading of another limb. In contrast, the roll and pitch are observable with inertial measurements thanks to the measurement of the gravity force. Thus, we can analyze the roll and pitch of one leg link independently of the roll and pitch of another leg link.

### 2.2. Biomechanical Analysis of the Tilt Angles

For completeness, we present a summary of our analysis of the tilt angles of an inertial localization system [22]. This analysis was first proposed for single-IMU localization systems. However, the results are extensible to inertial localization systems based on more than one inertial sensor.

Anatomical studies show that human motion is constrained [21]. In the case of inertial localization systems, the Euler angles are the signals that inform about human motion. More specifically, the roll and pitch measure the motion of the body limb where the inertial sensor is mounted and we can analyze these angles thanks to the experiment described in Section 2.1.

**Figure 4:**
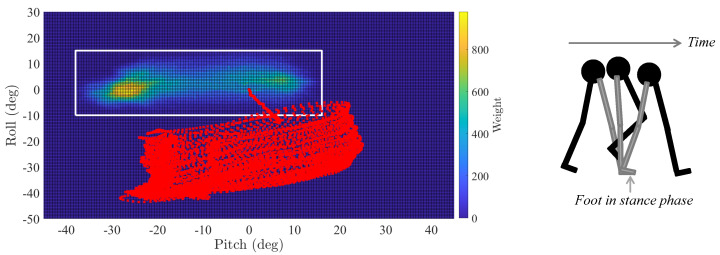
(**Left**) Heat map of the comfort zone (white rectangel) of the thigh. The red marks are the roll and pitch estimated by the inertial localization system based on a thigh-mounted inertial sensor [25]. (**Right**) Evolution of the right leg—in grey—during the stance phase.

Our analysis revealed that the pairs roll-pitch of a leg link are distributed around a constrained area, which we refer to as comfort zone, see the white rectangle in Figure 4. The comfort zone of a leg link tells us that not only is its motion constrained, but also that the leg links of different people move in a similar way regardless of their height, weight and walking style. It is also interesting to observe that the comfort zone of the upper thigh is larger than the comfort zone of the foot, which is expected since the range of motion of the thigh while walking is larger than the range of motion of the foot.

We use the comfort zone of each leg link to generate a probabilistic model of the roll and pitch of each leg link. More specifically, we model the roll and pitch of both the thigh and the foot during the stance phase of the foot as Gaussian distributions. Thanks to the maximum likelihood estimation (MLE) [26], we estimate the mean and standard deviations that best model the angles. These parameters are shown in Table 2. The advantage of Gaussian distributions is that they are easily integrated into Kalman filters [27], which is the algorithm through which we implement our inertial localization system [3].

It is important to highlight the consequences of our findings:

1. We can observe errors in inertial localization systems by analyzing human motion.
2. The errors in the Euler angles estimation of the inertial localization systems translate into incoherent motion of the leg links where the inertial sensor is mounted, see the red marks in Figure 4.
3. To analyze if an inertial localization system is estimating incoherent motion, we do not need the true trajectory. We need only to statistically characterize human motion and compare the estimated angles to the expected ones, for example, the probability density functions (PDFs) of the roll and pitch angles.

Our approach in Reference [22] is extensible to multi-IMU localization systems because their estimation of the roll and pitch of each leg link is the same as the one of a single-IMU localization system.

### 2.3. Biomechanical Analysis of the Relative Heading

The approach of the previous section is not applicable to observe the errors in the heading estimation. The reason is because the true heading cannot be estimated only with inertial measurements [24].

We propose to analyze the heading difference between the thigh heading and the foot heading in order to observe the errors in the heading estimation. The reason is that the heading estimation represents the direction of the walk which intuitively should be the same for both leg links. To analyze if our intuition is valid, we define the relative heading (*ψ_r_*) as the difference between the thigh heading (*ψ_t_*) and the foot heading (*ψ_f_*):

(1) $$\psi_{r}=\psi_{t}-\psi_{f}.$$

**Figure 5:**
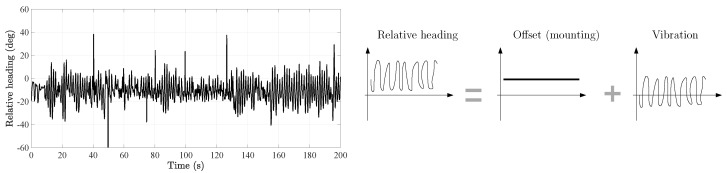
(**Left**) Relative heading between the thigh and foot as defined by Equation (1). (**Right**) Decomposition of the relative heading in its two components. This model is based on the data recorded during the motion tracking experiment in Section 2.1.

Figure 5 plots the ground truth relative heading between the thigh and the foot for one of the users that participated in the experiment of Section 2.1. In this figure, we observe that the ground truth relative heading has two components: a constant value or offset and vibrations. The constant value is due to the mounting of the sensors on the thigh and foot because the associated sensor frames were not perfectly aligned, see Figure 1. The vibrations around the offset are due to the motion of the leg links, that is, thigh and foot, during the walk.

All in all, we consider that our intuition is correct: the relative heading of two leg links is approximately the same. Yet, our experiment reveals that there are components of the relative heading that are not intuitive, for example, the effect of the mounting of the sensing devices on the leg links. These components of the relative heading are only observable when carrying out experiments like the one in presented in Section 2.1. That is, an experiment where devices that can measure absolute headings are used.

**Figure 6:**
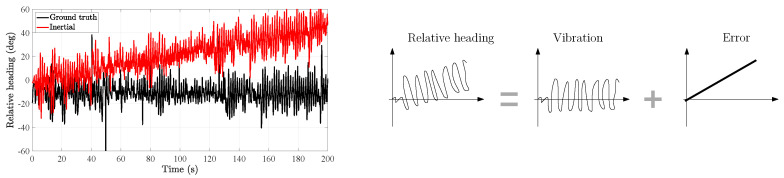
(**Left**) Relative heading between the thigh and foot, as defined by Equation (1), estimated with both the ground truth and the inertial data. (**Right**) Decomposition of the inertial relative heading in its two components. This model is based on the inertial measurements recorded during the motion tracking experiment in Section 2.1.

Figure 6 shows the relative heading estimated with both the ground truth data from the motion tracking system and the heading estimations of the inertial localization systems based on the thigh-mounted and foot-mounted inertial sensors. It is interesting to compare both estimations: while the ground-truth relative heading remains constant over time, the inertial relative heading increases linearly over time. In order to understand the biomechanical implications of Figure 6, let us imagine a user walking on a straight line, for example, with an initial heading of 0°. According to the inertial relative heading, while the user is walking on a straight line, the user’s thigh and foot are constantly rotating with respect to each other around the z-axis. This fact is not only false but also impossible from a biomechanical perspective because most users cannot rotate their thigh and foot as much as the inertial relative heading indicates in Figure 6.

Figure 6 shows also a simplified model of the inertial relative heading. In contrast to the model of the ground truth relative heading, the model of the inertial relative heading includes an error component, which is the difference between the heading drift of both the thigh and the foot. To analyze this error component, let us write the heading estimation of the inertial localization systems as follows:

(2) $$\psi_{t}=\psi \psi \psi_{vt}\psi \psi_{et},$$

(3) $$\psi_{f}=\psi \psi \psi_{vf}\psi \psi_{ef},$$

where *ψ* is the user’s true heading, *ψ_vi_* is the vibration in the heading due to the motion of the leg link during the walk and *ψ_ei_* is the heading drift due to the attitude tracking algorithm. The subindex *i* refers to the thigh (*t*) or the foot (*f*). By subtracting the two equations above, the relative heading can be expressed as:

(4) $$\psi_{r}=\left(\psi_{vt}-\psi_{vf}\right)+\left(\psi_{et}-\psi_{ef}\right),$$

where each addend represents each of the components on the right-hand picture of Figure 6, respectively.

In the inertial relative heading, we only observe the difference in the heading drift of the thigh and the foot, namely $\psi_{et}-\psi_{ef}$. Therefore, the relative heading allows us to perceive the error difference between the drift of the thigh heading and the drift of the foot heading. In general, both drifts are different and therefore the second addend in Equation (4) is non-zero, which allows us to observe that two inertial localization systems are estimating an incoherent heading.

Depending on the independent drifts, namely *ψ_et_* and *ψ_ef_*, the incoherent behaviour of the inertial relative heading will be more or less highlighted. More specifically, the higher the difference in the heading drifts, the steeper the slope of the inertial relative heading, see Figure 6. This slope is an incoherence from the biomechanical point of view as it means that the thigh and foot are constantly rotating with respect to each other while the user is walking.

To sum up, the relative heading is a new magnitude that represents the heading difference between the heading estimations of two inertial sensors mounted on two different leg links. Thanks to the relative heading, we can observe not only the heading errors but also that the heading error leads to a biomechanical incoherent motion. The limitation of our approach is that we can observe the difference in the heading errors but we are not able to estimate the heading drift associated to the thigh heading and the foot heading.

### 2.4. Proposed Tight Coupling System

This section presents the implementation of the tight coupling system, which takes as inputs the raw measurements of the thigh and foot inertial sensors and outputs the 3D-position of the user. The key feature of the tight coupling system is the integration of biomechanical constraints that guarantee that the Euler angles estimation of the tight coupling system is coherent with respect to human motion. By guaranteeing the coherence of the Euler angles, the biomechanical constraints are directly and indirectly influencing the accuracy of the position estimation of the tight coupling system.

**Figure 7:**
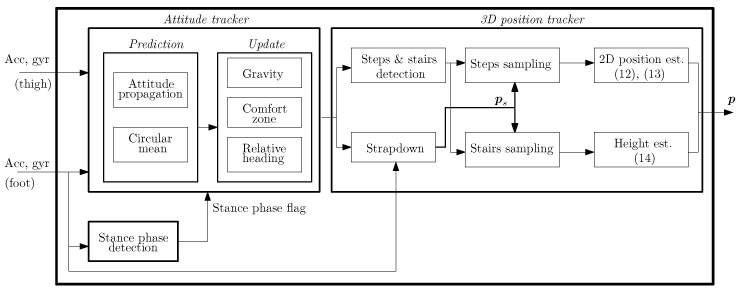
Block diagram of the tight coupling system. The inputs are the acceleration vector (*acc*) and the turn rate vector (*gyr*) of both the thigh inertial sensor and the foot inertial sensor. The output is the 3D-position of the user (***p***) and *est.* stands for estimation.

The block diagram of the tight coupling system is presented in Figure 7 and it consists of three main blocks: the *stance phase detection* block, the *attitude tracker* block and the *3D position tracker*. The *stance phase detection* block detects when the foot is in stance phase, see Figure 4. The reader is referred to Reference [3] and references therein for the description of stance phase detection algorithms. The detection of the stance phase is necessary to apply the constraints of the tilt angles, see Section 2.2.

The *attitude tracker* estimates the Euler angles of both the thigh and the foot. This block takes as input the raw data of both inertial sensors as well as the stance phase flag. This block also integrates the biomechanical constraints derived from the study in Section 2.2 and Section 2.3. Finally, the *3D position tracker* block estimates the user’s position by exploiting characteristic features from both the thigh-mounted and the foot-mounted sensors.

The next sections detail the implementation of the *attitude tracker* block and the *3D position tracker* block.

#### 2.4.1. Attitude Tracker

The attitude tracker implements an unscented Kalman filter (UKF) [28], which is suitable for processes that are non-linear, like the tracking of the attitude of a body [29]. The states vector (*x*) of our attitude tracker comprises the following elements: the attitude vector of the thigh (**Ψ***_t_*) the gyroscope bias vector of the thigh-mounted inertial sensor (***b**_**g**t_*), the attitude vector of the foot (**Ψ***_f_*), the gyroscope bias of the foot-mounted inertial sensor (***b**_**g**f_*) and the user’s heading (*ψ_u_*):

(5) $$x={\left[\Psi_{t}^{T},{b_{g}}_{t}^{T},\Psi_{f}^{T}{b_{g}}_{f}^{T},\psi_{u}\right]}^{T},$$

where ${\left(\cdot \right)}^{T}$ indicates the transpose operation. It is worth highlighting that all vector magnitudes are column vectors and that the user’s heading is a scalar.

Similarly to all Kalman filters, the UKF has two stages, namely the prediction and the measurement update, see Figure 7. During the prediction, the states vector is propagated following the process model. In our case, the process model to estimate both the attitude vectors and the gyroscope bias vectors is detailed in Reference [3] and references therein. In the case of the user’s heading (*ψ_u_*), it is estimated as the circular mean [30,31] between the thigh heading (*ψ_t_*) and the foot heading (*ψ_u_*):

(6) $$\psi_{u}=\arctan\left(\frac{\sin\left(\psi_{t}\right)+\sin\left(\psi_{f}\right)}{\cos\left(\psi_{t}\right)+\cos\left(\psi_{f}\right)}\right).$$

The main advantage of the circular mean is that it is better suited to estimate the average of circular magnitudes like angles, in this case, the average of the thigh heading and the foot heading.

The second stage of the UKF is the measurement update, during which some states are corrected with external measurements. In our case, the attitude tracker block implements three measurement updates: the gravity update, the comfort zone update and the relative heading update. The gravity update corrects the tilt angles of both leg links, namely the thigh and foot, when these are static or quasi-static. The reader is referred to Reference [3] and references therein for more details on the implementation of the gravity update.

In the following, we focus on the description of the implementation of the comfort zone update and the relative heading update since these are the result of the biomechanical study presented in this article.

##### Comfort Zone Update

The goal of the comfort zone update is to ensure that the tilt angles estimation of the attitude tracker is coherent with respect to the motion of the leg link. The motion of each leg link, namely the thigh and foot, is characterized by the Gaussian distributions of the comfort zone of each tilt angle of both the thigh and foot during the stance phase.

The knowledge provided by the Gaussian distributions is integrated in the UKF as soft constraints [32]. A soft constraint is a measurement update in which a state is expected to take a certain value which has an associated confidence. In our case:

- the states are the roll and pitch of both the thigh and foot,
- the expected values are the mean of the associated Gaussian distributions,
- the confidence values are the standard deviations of the associated Gaussian distributions.

Following the approach already proposed in Reference [22], the comfort zone update is applied upon the detection of the stance phase. The measurement vector (***z_c_***) is the following:

(7) $$z_{c}=\left[\phi_{t}^{Z},\theta_{t}^{Z},\phi_{f}^{Z},\theta_{f}^{Z}\right],$$

where $\phi_{t}^{Z}$, $\theta_{t}^{Z}$, $\phi_{f}^{Z}$ and $\theta_{f}^{Z}$ are the average values of the roll (*ϕ*) and pitch (*θ*) of the thigh (*t*) and foot (*f*), respectively, given in Table 2. The covariance matrix associated to the measurement vector (*z_c_*) is a diagonal matrix where the elements in the diagonal are the variance of each of the Gaussian distributions given by Table 2.

##### Relative Heading Update

The goal of the relative heading update is to ensure that the relative heading between the thigh and the foot remains coherent with respect to human motion, which means that the relative heading between the thigh and the foot should not increase over time, see Figure 6. Let us model the relative heading (*ψ_r_*) as a first-order linear regression over time (*t*):

(8) $$\psi_{r}=a_{r}\cdot t+b_{r}.$$

There are two main parameters that define the behaviour of the relative heading over time, namely the slope $a_{r}$ and the offset $b_{r}$. According to Section 2.3, the offset $b_{r}$ is given by the mounting of the sensors on the leg links. This offset cannot be estimated with inertial measurements because inertial measurements do not allow for the estimation of the absolute heading [24].

The slope $a_{r}$ is, however, known: it should be zero. Otherwise, the relative heading would indicate that the thigh and foot are constantly rotating with respect to each other over time, which is not true, see Section 2.3. We consider this expected value of the slope $a_{r}$ to be a pseudo-measurement; that is, a measurement that is not directly measured but known based on a physiological phenomenon [33]. The associated pseudo-measurement (***z_r_***) is as follows:

(9) $$z_{r}=a_{r}=0.$$

**Figure 8:**
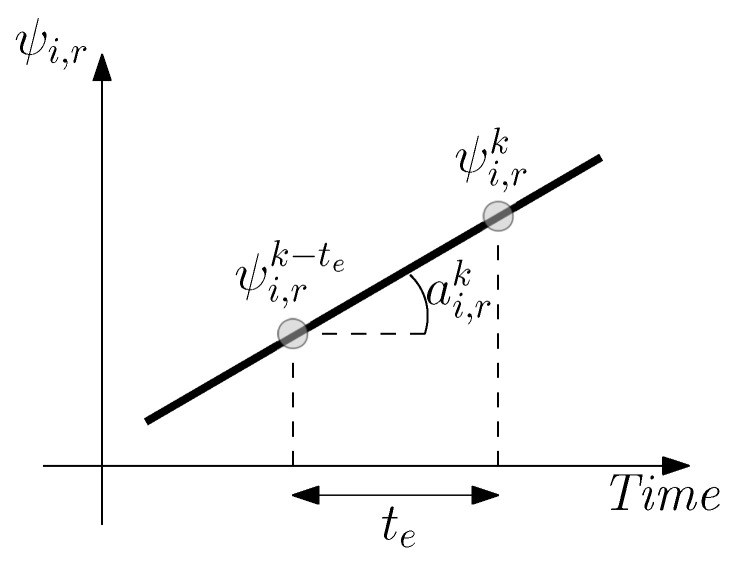
Visualization of the estimation of the slope of the relative heading for the *i*-th sigma point.

For the implementation of the relative heading update, we need to sample the slope of each of the sigma points of the UKF to then estimate a weighted average of the slope of each sigma point [28]. The estimated slope of the *i*-th sigma point at the *k*-th time ($a_{r,i}^{k}$) is calculated as:

(10) $$a_{r,i}^{k}=\frac{\psi_{r,i}^{k}-\psi_{r,i}^{k-t_{e}}}{t_{e}},$$

where $\psi_{r,i}^{k}$ is the relative heading of the *i*-th sigma point, with i={1,2,…,2n+1}, at the *k*-th time, *n* is the number of states of the UKF, that is, n=13, and $t_{e}$ is the elapsed time. In the practical implementation, continuous time is transformed to samples taking into account that the sampling frequency of the inertial sensors is 100 Hz. Figure 8 depicts the estimation of the slope $a_{r,i}^{k}$.

The relative heading of each sigma point ($\psi_{r,i}^{k}$) is estimated as the average over the last $t_{w}$ seconds of the difference between the thigh heading ($\psi_{t,i}^{k}$) and the foot heading ($\psi_{f,i}^{k}$) of the *i*-th sigma point, that is:

(11) $$\psi_{r,i}^{k}=\frac{1}{t_{w}}\cdot \sum_{j=k-t_{w}}^{k}\left(\psi_{t,i}^{k}-\psi_{f,i}^{k}\right).$$

For the implementation of the relative heading update, it is necessary to set the parameters $t_{e}$ and $t_{w}$. In this article, the value of these parameters is heuristically determined and set to 1 s and 10 s, respectively. These parameters indicate that the *attitude tracker* block applies the relative heading update every second ($t_{e}$ = 1 s). During this update, the relative heading is estimated as the average over the last 10 s ($t_{w}$ = 10 s) of the difference between the thigh heading and the foot heading.

The last parameter to be set in order to apply the relative heading update is the covariance of the pseudo-measurement. In this case, the pseudo-measurement is a perfect measurement which means that its covariance is zero [32]. However, setting the covariance of perfect measurements to zero has caused malfunctions in other works [34]. Therefore, we decide to set the covariance of the relative heading update to 4°, which is a value that we determine heuristically.

#### 2.4.2. 3D Position Tracker

The *3D position tracker* in Figure 7 estimates the 3D-position of the pedestrian based on the following information: the 3D-acceleration of the foot, the attitude of the foot inertial sensor, the stance phase detection flag and the pitch angle of the thigh.

**Figure 9:**
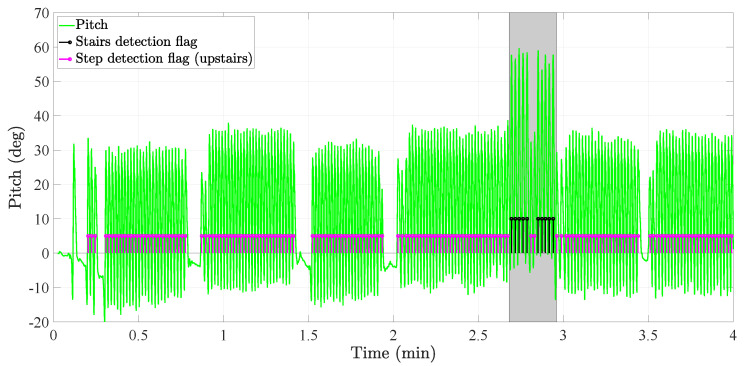
Thigh pitch over time. The steps and stairs flags are also plotted. These flags are generated based on the analysis of the amplitude of the thigh pitch.

**Figure 10:**
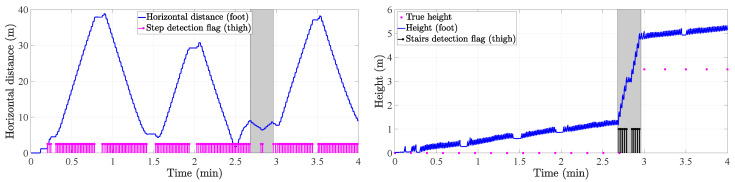
Norm of the horizontal distance (**left**) and height profile (**right**) estimated by the *strapdown* block of the tight coupling system. The grey area indicates when the user was walking the stairs.

More specifically, the *3D position tracker* follows the next steps:

1. in the *strapdown* block, the strapdown algorithm is implemented. This algorithm double-integrates the 3D-acceleration of the foot to estimate the pedestrian’s 3D position. Prior to the double-integration, the 3D-acceleration of the foot has to be projected onto the navigation frame, see Figure 1. This projection is done with the attitude of the foot IMU. Additionally, the *strapdown* block implements the Zero Velocity UpdaTe (ZUPT) to correct the velocity, and therefore the pedestrian’s 3D-position, upon the detection of the foot stance phase. For more details on the implementation of the strapdown block, the reader is referred to References [3,29].
2. in the *steps & stairs detection* block, horizontal steps as well as vertical steps, that is, steps walking upstairs and steps walking downstairs, are detected. This detection is done by analyzing the amplitude of the thigh pitch as well as its maximum and minimum values [25]. An example of the thigh pitch while a user is walking on a flat surface as well as upstairs is given in Figure 9. We can clearly observe how the thigh pitch is different regarding its amplitude, maximum and minimum while walking on a flat surface than while walking the stairs.
3. It samples the output of the *strapdown* block ($p_{s}$) upon the detected steps to estimate the horizontal displacement (*s*) and vertical displacement (*v*) between consecutive steps or stairs as follows:
  - the horizontal displacement (*s*), or step length, is estimated as $s^{k}=\left|p_{s,xy}^{k}-p_{s,xy}^{k-1}\right|$, where *k* is the step or stair index, $p_{s,xy}$ is the horizontal position vector estimated by the strapdown block and |·| denotes the norm of the argument. An example is given in Figure 10. This figure shows the norm of the horizontal distance and the step detection flags.
  - the vertical displacement (*v*) is estimated as $v^{k}=p_{s,z}^{k}-p_{s,z}^{k-1}$, where *k* is the step or stair index and $p_{s,z}$ is the z-component, or height, estimated by the strapdown block. An example is given in Figure 10. The vertical displacement is estimated only when the user is walking the stairs.
4. Finally, the tight coupling system estimates the 3D-position of the pedestrian (p) as follows:
  (12) $$\begin{array}{ccc} p_{x}^{k} & = & p_{x}^{k-1}+s^{k}\cdot \sin\left(\psi_{u}\right), \end{array}$$
  (13) $$\begin{array}{ccc} p_{y}^{k} & = & p_{y}^{k-1}+s^{k}\cdot \cos\left(\psi_{u}\right), \end{array}$$
  (14) $$\begin{array}{ccc} p_{z}^{k} & = & p_{z}^{k-1}+v^{k}, \end{array}$$
  where $p^{k}=\left(p_{x}^{k},p_{y}^{k},p_{z}^{k}\right)$, *k* is the step or stair index and, as previously indicated, $\psi_{u}$ is the user’s heading estimated by the *attitude tracker* block. These equations are implemented in the *2D position est.* and *Height est.* blocks in Figure 7.

The aforementioned technique of sampling the output of the strapdown upon the stairs detection has successfully been implemented in Reference [35]. In this article, we extend this approach by sampling not only the z-component of the position vector ($p_{s}$) upon the detected stairs but also the horizontal position vector upon the detected steps. By implementing the sampling of the position vector ($p_{s}$), we aim at reducing the error accumulation due to the integration of the errors in the signals that are inputs to the *strapdown* block. For instance, this error accumulation is clearly observed in the height profile of Figure 10 by comparing the true height to the height estimated by the *strapdown* block.

## 3. Results

In this section, we analyze two different aspects of this work. Firstly, we evaluate the effect that the motion constraints have in the estimation of the Euler angles of both the thigh and the foot. Secondly, we evaluate the performance of the tight coupling system and compare it to the performance of two state-of-the-art inertial localization systems based on only one inertial sensor.

### 3.1. Evaluation of the Coherence of the Euler Angles

This section divides the evaluation of the Euler angles into two parts: the evaluation of the tilt angles, that is, roll and pitch, and the evaluation of the relative heading.

#### 3.1.1. Evaluation of the Tilt Angles

In the case of the tilt angles, we want to evaluate the effect of the comfort zone update on the estimation of both the roll and pitch. To that end, we will assess the cumulative distribution function (CDF) of the tilt angles of the thigh and foot before and after applying the comfort zone update.

We use the data collected from the motion tracker during the motion tracking experiment, see Section 2.1, to estimate a reference CDF of the tilt angles of the thigh and foot. Then, we make use of the residual sum of squares (RSS) to compare the reference CDF to the CDF of the tilt angles estimated by the tight coupling system. The RSS is frequently used in statistics to compare the discrepancy between a data set and a model [36]. Thus, the larger the RSS the more different the data set and the model are and therefore, the less accurate the model fits the data set. The RSS ($r_{ss}$) is estimated as follows:

(15) $$r_{ss}=\sum_{i=1}^{n}{\left(y_{i}-f\left(x_{i}\right)\right)}^{2},$$

where $y_{i}$ is the *i*-th sample of a data set with *n* samples, $x_{i}$ is the observation and $f(x_{i})$ represents the model that estimates $y_{i}$. In our particular case, $y_{i}$ is given by the reference CDF and $f(x_{i})$ is given by the estimated CDF.

**Figure 11:**
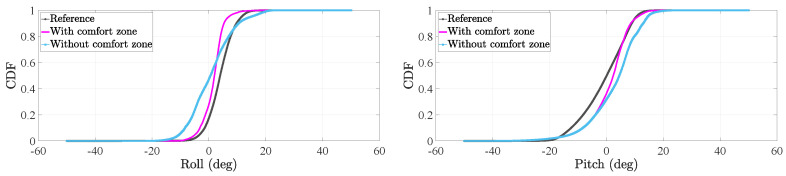
Cumulative distribution function (CDF) of the tilt angles of the thigh inertial sensor during the stance phase.

**Figure 12:**
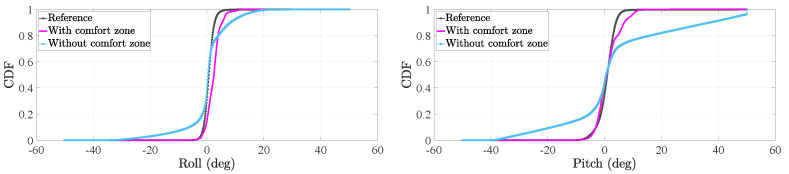
CDF of the tilt angles of the foot inertial sensor during the stance phase.

Figure 11 and Figure 12 show the CDFs of the tilt angles of the thigh and foot respectively. In these plots, we can visually compare the CDF of the tilt angles estimated by the tight coupling system to the respective reference CDFs. We can clearly observe how the CDF of each tilt angles is closer to the reference CDF after applying the comfort zone update.

Table 3 present the RSS of all the distributions in Figure 11 and Figure 12. Both tilt angles of the thigh inertial sensor benefit from the comfort zone update. This fact is indicated in Table 3 by RSS values that are smaller when the comfort zone is applied than when it is not applied in the tight coupling system.

In Table 3, we can see how the comfort zone update improves the distribution of the foot pitch significantly. In fact, the RSS is reduced from 16.4 to 0.8. This reduction is reflected in Figure 12 by an estimated CDF that is significantly close to the reference one when the comfort zone update is applied.

In the case of the foot roll, we observe that the RSS increases slightly from 3.0 to 3.9. When we analyze the associated plot in Figure 12, we see that the CDF without the comfort zone models almost perfectly the reference distribution around 0° but around the elbows of the CDF, the estimated CDF models the reference CDF less accurately. In contrast, the CDF of the roll after applying the comfort zone update models better the reference CDF around its elbows than around 0°. The reason is that, during the comfort zone update, the foot roll is corrected to a value of 0.8° instead of 0°, which seems to be the value of the foot roll without the comfort zone update according to the true CDF of the foot roll. Since the range of values of the foot roll is smaller than the range of values of the foot pitch, small shifts in the angle values are more noticeable for the foot roll than the foot pitch. Therefore, provided the results of Table 3, one could decide not to implement the comfort zone update of the foot roll.

**Figure 13:**
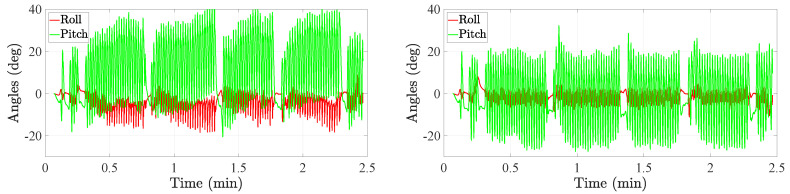
Tilt angles of the thigh before the comfort zone update (**left**) and after the comfort zone update (**right**).

Figure 13 shows the tilt angles of the thigh before and after the application of the comfort zone update. It is clearly observed that, when the comfort zone update is not applied, the attitude tracker estimates incoherent tilt angles. The incoherence is observed by the average values of both angles, which vary over time. However, after the comfort zone update is applied, both the roll and pitch become coherent with an average value that is approximately constant over time.

#### 3.1.2. Evaluation of the Relative Heading

We now evaluate the effect of the relative heading update in the relative heading between the thigh and the foot. To that end, we model the relative heading as a first-order linear regression, see Equation (8). Then, we estimate the parameters of the model, that is, the slope $a_{r}$ and the offset $b_{r}$ of the relative heading of each walk. Finally, we compare the slope of the relative heading before and after applying the relative heading update.

For this evaluation, we use the walks presented in Reference [37]. The reason why we choose these walks are because they are longer, that is, at least 15 min each. Therefore, they will allow us for an appropriate estimation of the parameters of the first-order linear regression model. It is important to highlight that the reference slope is zero, as indicated in Section 2.3.

**Figure 14:**
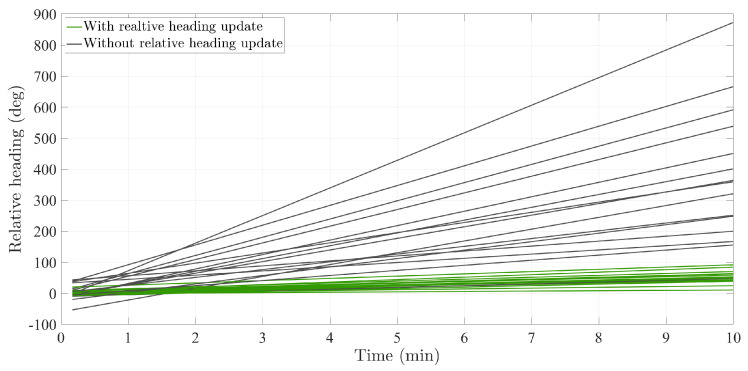
First-order linear regressions that model the relative heading before and after applying the relative heading update in the tight coupling system.

Figure 14 shows the first-order liner regressions for each of the walks before and after applying the relative heading update in the tight coupling system. We observe that the slope of the relative heading decreases after the application of the relative heading update. As expected, the relative heading of one walk drifts differently than the relative heading of a different walk. Nonetheless, it is clear from Figure 14 that the relative heading update improves the coherence of the relative heading by reducing its slope. In fact, if we estimate how much the slope of the relative heading is reduced in each walk and average these values, we see that the relative heading update reduces the slope of the relative heading in 78.5%. We consider this reduction a significant improvement in the performance of the relative heading of the tight coupling system.

**Figure 15:**
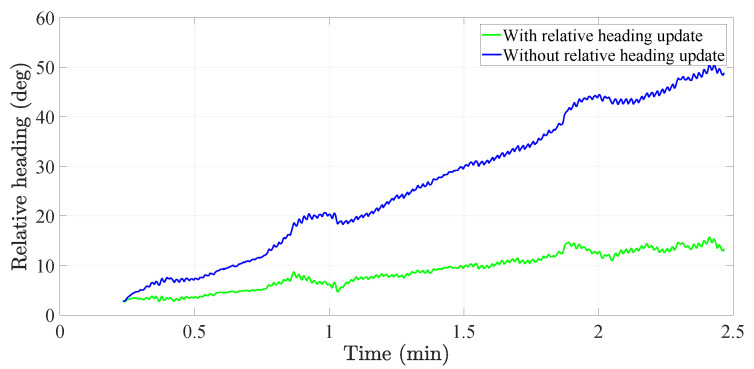
Comparison of the relative heading before and after the implementation of the relative heading update in the attitude tracker.

Figure 15 shows a comparison of the relative heading before and after the implementation of the relative heading update in the *attitude tracker* block of the tight coupling system. We can clearly observe that the relative heading grows much faster when the relative heading update is not applied than when it is applied.

### 3.2. Evaluation of the Tight Coupling System

In this section, we first present the ground truth system with which we will generate performance metrics to characterize the proposed tight coupling system. Then, we evaluate it and compare its performance to the ones of two state-of-the-art single-sensor inertial localization systems.

#### 3.2.1. Evaluation Methodology

To this day, there is no widely-adopted evaluation methodology for inertial localization systems [38]. Therefore, in this article, we use our own evaluation methodology which has been presented in detail in Reference [37]. For completeness, we present a brief summary of our evaluation methodology in the following points:

- our ground truth is based in ground truth points with known location. These points are deployed throughout a five-storey building. We have measured the ground truth points with a laser distance measurer which has approximately centimeter accuracy. We follow the recommendation of the standard for evaluation of localization systems [39]. According to this standard, the accuracy of the ground truth systems should be at least one order of magnitude better than the expected accuracy of the systems under test. Provided that the state of the art systems have an accuracy in the order of meters [6], we consider our ground truth system to be accurate enough to evaluate the performance of the localization systems under test. It is worth highlight that a similar ground truth system has been used in indoor localization competitions [40].
- we use two metrics to evaluate the performance of the localization systems:
  – position error *e_p_*, which is defined as:
    (16) $$e_{p}=|p_{i}^{r}-p_{i}^{w}|,$$
    where $p_{i}^{r}$ is the true 2D-coordinate of the *i*-th ground truth point and $p_{i}^{w}$ is the position of the *i*-th ground truth point estimated by the localization system.
  – height error *e*_h_, which is defined as follows:
    (17) $$e_{h}=\frac{\left|h_{i}^{r}-h_{i}^{w}\right|}{{\Delta h}_{i}^{r}},$$
    where $h_{i}^{r}$ and $h_{i}^{w}$ are the true height and the estimated height of the *i*-th ground truth point, |·| denotes the absolute value of the argument. Finally, $\Delta h_{i}^{r}$ is the total height change at the *i*-th ground truth point and it is estimated as follows:
    (18) $${\Delta h}_{i}^{r}=\sum_{j=0}^{i}\left|h_{j}-h_{j-1}\right|,$$
    where *h_j_* is the height of the *j*-th ground truth point. The height error in Equation (17) is normalized to the change in height, which means that, for instance, a height error *e*_h_ of 0.1 m/m tells us that the makes an error of 10 cm in a height change of 1 m.
- In order to identify when the volunteer reaches a ground truth point, we follow a similar strategy to that implemented in indoor localization competitions [40]. This strategy comprises two steps:
  – We designed the trajectories a priori by defining the sequence in which the volunteer should visit the ground truth points.
  – We instructed the volunteer to stop for 2–3 s on each ground truth point. Then, we detected that a volunteer was at a ground truth point by analyzing the norm of the acceleration and turn rate vector of either the thigh-mounted or the foot-mounted inertial sensor.
- the data set is summarized in Table 4

#### 3.2.2. Results and Discussion

In the following, we evaluate three inertial localization systems:

- the tight coupling system, which is the system proposed in this article and whose performance we want to quantify,
- two state-of-the-art systems based on a single inertial sensor each. These systems are the reference against which we want to compare the performance of our proposed tight coupling system, namely:
  – the thigh inertial localization system, which is an inertial localization system based on a thigh-mounted inertial sensor [25], see Figure 1, and
  – the foot inertial localization system, which is an inertial localization system based on a foot-mounted inertial sensor, see Figure 1 [3].

Table 5 summarizes different statistics of the performance metrics of each of the inertial localization systems under test. In addition to the mean and standard deviation of the 2D-position and height error, we also include the third quartile of both errors. The third quartile of an error is commonly used in inertial localization competitions to compare the performance of the competing systems [40]. The third quartile of an error means that the respective error is equal to or less than a given value.

**Figure 16:**
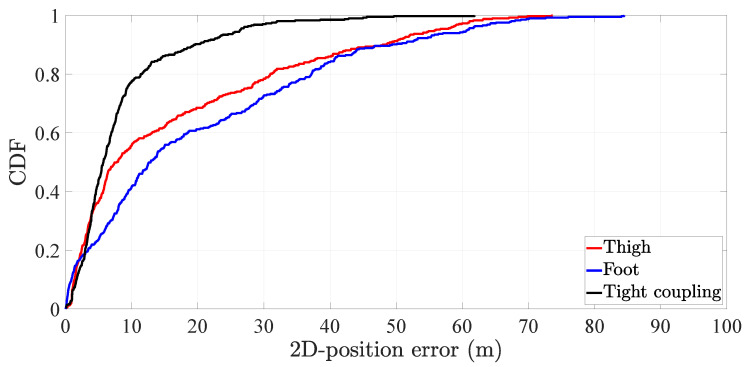
CDF of the 2D-position error of the tight coupling system and the single-sensor inertial localization systems based on a thigh-mounted and a foot-mounted inertial sensor.

The tight coupling system reduces the average 2D-position error in 50% with respect to the thigh inertial localization system and 58.4% with respect to the foot inertial localization system. The third quartile, however, shows a greater performance improvement of the tight coupling with respect to both inertial localization systems. More specifically, the third quartile of the tight coupling systems outperforms in 66% and 72% the third quartile of thigh and the foot inertial localization systems respectively. The performance improvement of the tight coupling over the single-sensor inertial localization systems is also observed in the CDF of the 2D-position error, see Figure 16.

The performance improvement of the tight coupling system over the single-IMU systems is due to the integration of biomechanical constraints within the position estimation process. We believe that this performance improvement is an indication that our approach of incorporating knowledge of human motion into the inertial localization system to improve the accuracy of the position estimation is successful and worth further investigation. It is important to highlight that, in our proposal, the knowledge on human motion is incorporated through the Euler angles of the leg links and that these angles influence then the position estimation.

**Figure 17:**
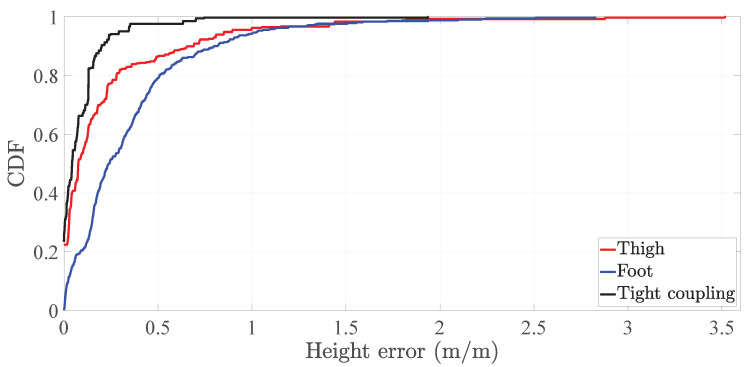
CDF of the height error of the tight coupling system and the single-sensor inertial localization systems based on a thigh-mounted and a foot-mounted inertial sensor.

On the average height error, Table 5 shows that the tight coupling system outperforms in 87.5% and 75% the thigh and the foot inertial localization systems, respectively. Nonetheless, this improvement is less if we assess the third quartile of the height error, which shows that the tight coupling system outperforms the thigh inertial localization system only in 47.8% although the improvement with respect to the foot inertial localization system remains approximately similar, namely 74%. Figure 17 depicts how the CDF of the height error of the tight coupling system outperforms the CDFs of the height error of both the reference systems.

Our proposed tight coupling system combines two approaches to reduce the height error:

1. The improvement in the coherence of the thigh pitch thanks to the comfort zone update [22]. This approach allows the thigh inertial localization system to better detect the stairs and therefore, to estimate a vertical displacement only when necessary.
2. The stairs sampling of the z-component of the position estimated by the strapdown algorithm, see Figure 7 [35]. This approach addresses the error accumulation in the z-component by estimating a vertical displacement from the z-component of the position only when the user is walking the stairs.

Now, we would like to analyze an example trajectory of the tight coupling system and compare it to the trajectories estimated by the state-of-the-art systems. Prior to doing so, we would like to comment on an important fact of the evaluation of inertial localization systems: the duration of the walks. Our data set comprises walks that last at least 15 min. Provided that inertial localization systems accumulate errors over time [29], the longer the walk used to evaluate an inertial localization system is, the higher the average position error will be at the end of a trajectory. The duration of the walks in this work is 3-5 times higher than the average duration of past works where we characterized the performance of the thigh and foot inertial localization systems [5,6]. Therefore, it is expected that the errors of these systems are higher in this work than the error values obtained in past works. An alternative to address the time-dependency of the proposed performance metrics is to normalize the 2D-position error with respect to the walked distance. However, such an approach would need another type of ground truth system which is out of the scope of this work.

**Figure 18:**
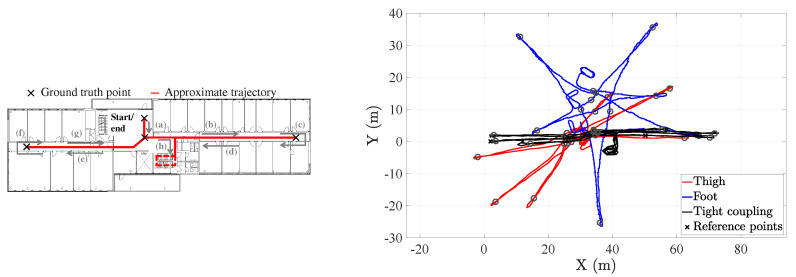
(**Left**) Approximate true trajectory. (**Right**) Odometry estimated by the inertial localization systems under test. The circle marks are the position of the ground truth points estimated by the inertial localization systems under test. This figure shows the first 5 min of the walk.

Figure 18 shows an example odometry of the first 5 min of one of the walks. By comparing the estimated trajectories to the true trajectory, we can clearly see that the tight coupling system is outperforming both the thigh and the foot inertial localization systems. In fact, we can see how the heading of both state-of-the-art inertial localization systems drift over time, which causes the estimated trajectory to drift counterclockwise in this particular example. In contrast, the heading of the tight coupling system has barely drift and therefore the estimated trajectory resembles the true trajectory.

**Figure 19:**
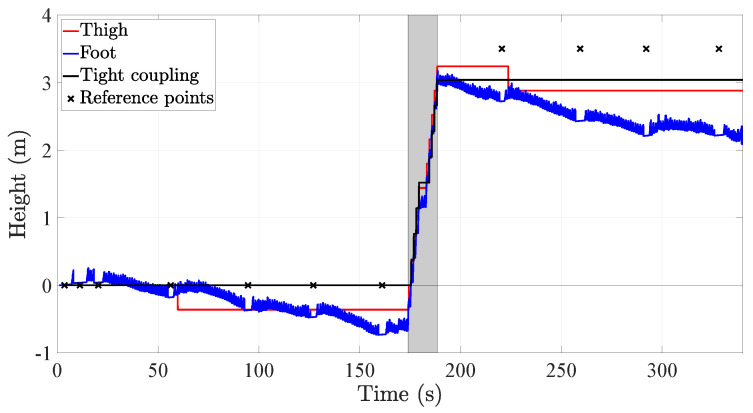
Height profile estimated by the inertial localization systems under test. For visualization purposes, we show only the first change in floors, namely from the initial floor (**second floor**) to the next one (**third floor**).

Figure 19 shows the height profile estimated by the inertial localization systems. There are several elements to highlight in this plot. Firstly, the height profile of the foot inertial localization system drifts over time, which causes the height to increase even if the volunteer was walking on a flat surface. Secondly, the height profile of the thigh inertial localization system is affected by the outliers in the detection of the stairs, for example, at 59 s and 223 s. Finally, the tight coupling system is affected by neither of the aforementioned problems. However, we see that the height change does not reach the true height of the ground truth points on the third floor. The reason is that while walking the stairs, the volunteer walked the first and last stairs with the leg that did not have the inertial sensors. Therefore, these two stairs are not detected by the tight coupling system and, thus, are not accounted for in the estimation of the height profile.

Our tight coupling system offers an alternative for drift-compensation techniques that is based on the use of only inertial technology. The tight coupling system relies on the relative heading to observe the error difference between the error of the thigh heading and the foot heading. Our proposal differs radically from the state-of-the-art, where drift-compensation techniques rely on either using a map, landmark detection or SLAM algorithms. The former technique relies on the availability of a map and the latter two techniques require the total or partial repetition of a trajectory to compensate for the heading drift. In our proposal, we depend on neither of these two requirements to compensate for the heading drift.

## 4. Conclusions

In this article, we have presented the tight coupling system, which is an inertial localization system that simultaneously processes the inertial measurements of two inertial sensors placed on the upper thigh and on the front part of the foot. We have presented our analysis of the motion of the leg and we have derived a set of constraints from this analysis. These constraints have been integrated in the tight coupling system. Finally, we have assessed the performance of the tight coupling system and compared its performance to the one of two state-of-the-art inertial localization systems.

From our work, we derive three main conclusions. The first one is that, thanks to our study of the motion of the human leg, not only have we observed the errors in the tilt angles, but also in the relative heading between leg links, namely the thigh and the foot. The second conclusion is that, to the best of our knowledge, it is the first time that the heading errors are observed when only inertial measurements are used and without any external reference nor partial or total repetition of the trajectory. We have observed the heading errors through the definition of the relative heading. The limitation of our approach is that we cannot estimate the heading drift of each heading and that our approach does not work if both heading estimations drift in the same direction and by the same amount. The last conclusion is that we can improve the accuracy of the position estimation by introducing the biomechanical dimension in an inertial localization system, for example, by constraining the human motion derived from the Euler angles estimated by an inertial localization system.

## 5. Patents

The work presented in this work is related to a patent which is being under consideration at the German Patent Office. The file number is 10 2017 124 173.6.

## Figures and Tables

**Table 1:** Summary table of the experiments to evaluate the biomechanical motion of the leg.

Feature	Value or Description
**Number of users**	9
**Number of trajectories**	3
**Repetitions per trajectory**	2
**Total amount of data**	3 h 37 min
**Output data (motion tracking system)**	3D position and attitude
**Output data (IMU)**	3D acceleration and 3D turn rate

**Table 2:** Parameters of the maximum likelihood estimation (MLE) Gaussian distributions of each tilt angle of the thigh and the foot.

Variable	Mean μ [°]	Standard Deviation σ [°]
Thigh roll	4	4.5
Thigh pitch	−0.8	8
Foot roll	0.8	1.7
Foot pitch	0.8	3

**Table 3:** Residual sum of squares (RSS) of the tilt angles distribution of the thigh inertial sensor and the foot inertial sensor. The angles are taken only during the stance phase. The values of this table have no units since they represent a difference between probabilities.

System Description	Thigh		Foot	
	Roll	Pitch	Roll	Pitch
Without comfort zone	8.1	5.5	3.0	16.4
With comfort zone	4.5	2.1	3.9	0.8

**Table 4:** Summary of the experiments.

No. of Users	Total Time	Total No. of Ground Truth Points
10	10 h 20 min	482

**Table 5:** Summary of the statistics of the systems under test. Q${}_{3}\left(\cdot \right)$ stands for the third quartile of the argument.

System Description	2D-Position Error (*e_p_*) [m]	Q_3_ (*e_p_*) [m]	Height Error (*e*_h_) [m/m]	Q_3_ (*e*_h_) [m/m]
Tight coupling system (our proposal)	8.4±8.4	9.2	0.1±0.2	0.12
Thigh inertial localization system ([25])	16.6±17.9	27	0.8±2.0	0.23
Foot inertial localization system ([3])	20.2±19.0	32.7	0.4±0.4	0.46

## Abbreviations

The following abbreviations are used in this manuscript:

CDF	Cumulative Distribution Function
GNSS	Global Navigation Satellite System
IMU	Inertial Measurement Unit
MLE	Maximum Likelihood Estimator
SLAM	Simultaneous Localization And Mapping
PDF	Probability Density Function
RSS	Residual Sum of Squares
UKF	Unscented Kalman Filter
ZUPT	Zero-velocity UPdaTe
